# Assessment of Patient Satisfaction Using a New Augmented Reality Simulation Software for Breast Augmentation: A Prospective Study

**DOI:** 10.3390/jcm11123464

**Published:** 2022-06-16

**Authors:** Simone La Padula, Rosita Pensato, Francesco D’Andrea, Ludovica de Gregorio, Concetta Errico, Umberto Rega, Luigi Canta, Chiara Pizza, Giovanni Roccaro, Raphaelle Billon, Endri Dibra, Jean Paul Meningaud, Barbara Hersant

**Affiliations:** 1 Department of Plastic and Reconstructive Surgery, Università Degli Studi di Napoli Federico II, Via Pansini 5, 80131 Napoli, Italy; rositapensato@gmail.com (R.P.); profdandrea@gmail.com (F.D.); ludovicadegregorio95@gmail.com (L.d.G.); concettaerrico@me.com (C.E.); regaumberto93@gmail.com (U.R.); info@pierluigicanta.it (L.C.); 2 Department of Plastic, Reconstructive and Maxillo facial Surgery, Henri Mondor Hospital, University Paris XII, 51 Avenue du Maréchal de Lattre de Tassigny, 94000 Creteil, France; chiapiz@gmail.com (C.P.); dott.giovanniroccaro@gmail.com (G.R.); raphbillon@hotmail.com (R.B.); endri.dibra@arbrea-labs.com (E.D.); meningaud@me.com (J.P.M.); barbara.hersant@gmail.com (B.H.)

**Keywords:** augmented reality, 3D simulation, breast augmentation simulation, breast implants, breast augmentation outcome assessment, assessment scale, BREAST-Q

## Abstract

**Background:** Breast augmentation is one of the most frequently performed plastic surgery procedures. Providing patients with realistic 3D simulations of breast augmentation outcomes is becoming increasingly common. Until recently, such programs were expensive and required significant equipment, training, and office space. New simple user-friendly programs have been developed, but to date there remains a paucity of objective evidence comparing these 3D simulations with post-operative outcomes. The aim of this study is to assess the aesthetic similarity between a pre-operative 3D simulation generated using Arbrea breast simulation software and real post-operative outcomes, with a focus on patient satisfaction. **Methods:** The authors conducted a prospective study of patients requiring breast augmentation. Patients were asked to assess how realistic the simulation was compared to the one-year post-operative result using the authors’ grading scale for breast augmentation simulation assessment. Patient satisfaction with the simulations was assessed using a satisfaction visual analogue scale (VAS) ranging from 0 (not at all satisfied) to 10 (very satisfied). Patient satisfaction with the surgical outcome was assessed using the BREAST-Q Augmentation Module. **Results:** All patients were satisfied with the simulations and with the attained breast volume, with a mean VAS score of 8.2 ± 1.2. The mean simulation time took 90 s on average. The differences between the pre-operative and one-year post-operative values of the three BREAST-Q assessments were found to be statistically significant (*p* < 0.001). **Conclusions:** Three-dimensional simulation is becoming increasingly common in pre-operative planning for breast augmentation. The present study aimed to assess the degree of similarity of three-dimensional simulations generated using Arbrea Breast Software and found that the use of the software provided a very satisfying representation for patients undergoing breast augmentation. However, we recommend informing patients that only the volume simulation is extremely accurate. On the other hand, it is necessary to not guarantee an absolute correspondence regarding the breast shape between the simulation and the post-operative result.

## 1. Introduction

Nowadays, breast augmentation is one of the most common plastic surgery procedures performed all over the world. This procedure is based on the use of silicone implants of various shapes and sizes through different approaches. There is often a discrepancy between patient’s expectation and the outcome. This is one of the main causes of revision surgery. In most cases, starting with the first consultation, patients require a pre-operative simulation of the result. One of the critical steps of a satisfactory breast augmentation is the accurate choice of implants. The patient may indicate the desired size and shape of the implant, but the surgeon takes full responsibility for the final choice. Pre-operative planning involves testing several breast implants in order to choose the one that best suits the patient. This kind of simulation has been demonstrated to improve post-operative patient satisfaction overall and with respect to breast size [1,2,3,4,5,6,7,8]. Nevertheless, this kind of simulation may not be realistic, as sizers are applied in the bra over the breast, patients may not be wearing the right garments, and a large variety of implants may not be available (round or anatomical: XP, HP, MP) in the surgeon’s office. Moreover, the implant may undergo compression under the garments, leading to a non-realistic simulation. Furthermore, if a mastopexy is indicated, it is not possible to obtain a realistic post-operative simulation with breast sizers. These disadvantages have led to the development of 3D simulation software [6]. Arbrea Breast Software (ABS) is a 3D, live augmented reality simulation software for pre-visualization of breast surgery results that allows a more accurate prediction of the appropriate breast implant size and volume. Choosing the surgical approach, the implant shape, and the simulation of mastopexy in the case of breast ptosis is also possible through software analysis. Despite the vast potential of this program, only a few cases comparing the three-dimensional simulations and the relevant post-operative results have been published. The present study evaluates the extent to which ABS generates an accurate three-dimensional simulation of the actual post-operative results. This study aims to assess patient satisfaction with the use of ABS simulation in women with breast hypoplasia.

## 2. Material and Methods

The authors conducted a prospective study of patients requiring breast augmentation.

Inclusion criteria were an age of 18 years or older, BMI < 30, and breast hypoplasia; exclusion criteria were breast asymmetry, tuberous breast, ptotic breast, breast lesions, and BMI > 30.

Patients were informed about the topic and characteristics of our study and written informed consent was obtained from all participants. The study was conducted according to the guidelines of the Declaration of Helsinki. Ethical approval was given by the French institutional committee with the following judgment reference number: 2020-E0125-101. Arbrea Breast Software was introduced worldwide in 2018 to help plastic surgeons and patients choose the appropriate size and volume for their breast implants. Before 2018, the first author only used simulations with breast implant sizers under the bra. At the time of the second pre-operative visit, patients were sized using an implant in a larger bra. The desired implant was chosen after testing implants of different volume and shape.

Indications, the surgical procedure, and risks were then discussed with the patient. The whole process took one hour on average. Patients enrolled in this study underwent breast augmentation performed by the first author. These women had voluntarily sought a breast augmentation procedure that had been planned at least 1 month before the beginning of the study. Pre-operative mammogram and ultrasound (US) imaging were prescribed to all patients to exclude breast lesions. Pre-operative and one-year post-operative photographs were taken. Patients were asked to assess how realistic the simulation was compared to the one-year post-operative result using our validated grading scale (ranging from 0 to 4) for breast augmentation simulation assessment (Table 1). This scale was validated using the Rasch model, inter-rater reliability, and intra-rater reliability and exceeded all criteria for acceptability, reliability, and validity [8,9].

At a one-year follow-up appointment, patients were asked: “How satisfied are you with the volume of your breasts?” The assessment of the outcomes was carried out using a satisfaction visual analogue scale (VAS) ranging from 0 (not at all satisfied) to 10 (very satisfied). Patient satisfaction with surgical outcomes was assessed using the BREAST-Q Augmentation Module [10,11,12,13,14,15,16,17,18,19,20,21,22,23,24,25,26,27]. Pre-operative and one-year post-operative BREAST-Q scores were compared. The three BREAST-Q parameters assessed were patient satisfaction, psychological well-being, and sexual well-being. All of the authors took full responsibility for the integrity and confidentiality of the data. Analyses of continuous variables were conducted using a Student’s T-test. A *p*-value of less than 0.05 was considered statistically significant. The statistical analysis was performed using the software PRISM, version 7 (Graph Pad, San Diego, CA, USA).

### Arbrea Breast Software (ABS)

Arbrea Breast Software, developed by Arbrea Labs, a Swiss company based in Zurich, is a 3D, live augmented reality simulation software for pre-visualization of breast surgery. After three pictures of the patient were taken through the standard RGB back-facing camera of an iPad, the three-dimensional simulation of actual post-operative outcomes was performed using the artificial intelligence installed in the device (iPad) instead of on a server. This guarantees maximum privacy and speed to both the patient and the surgeon and enables the latter to run the software without the need to be connected to the internet. The results of 3D simulation are observed, along with measurements of the breasts, either through a 3D view based on three images (front and two lateral views), or live in augmented reality (AR) as directly projected onto the body of a moving patient. In this way the patient can appreciate the possible outcomes of various types of implants of different sizes and shapes from multiple and varied angles and views.

This software can also be successfully used for breast ptosis (mastopexy with implants).

## 3. Results

A total of 40 women who underwent breast augmentation between October 2018 and February 2020 were included in the study. The mean age of the patients was 29.5 ± 3.1 years (range, 18–53 years). Thirty-three patients had implants placed in a prepectoral plane, while seven patients underwent dual plane breast augmentation through an inframammary incision. The average implant volume was 320 mL (range, 250–400 mL). Integrity Sebbin’s semi-smooth, round, moderate-profile mammary implants were used. A semi-smooth implant is particularly soft to the touch, with skin-like qualities: thin, natural, and resistant. In all cases, the implants were irrigated intraoperatively with a solution containing 160 mg of gentamicin by piercing the implant case with a sterile needle. This procedure reduces the risk of contaminating the implant with germs and dust.

A 7-day post-operative antibiotics course was prescribed, and the use of a post-operative bra of appropriate size for 4 weeks was advised. No major complications were observed (Table 2).

All patients were very satisfied with the post-operative outcome at the one-year follow-up appointment. The differences between the pre-operative and one-year post-operative values of the three BREAST-Q assessments were found to be statistically significant (*p* < 0.001) (Table 3). 

**Figure 1:**
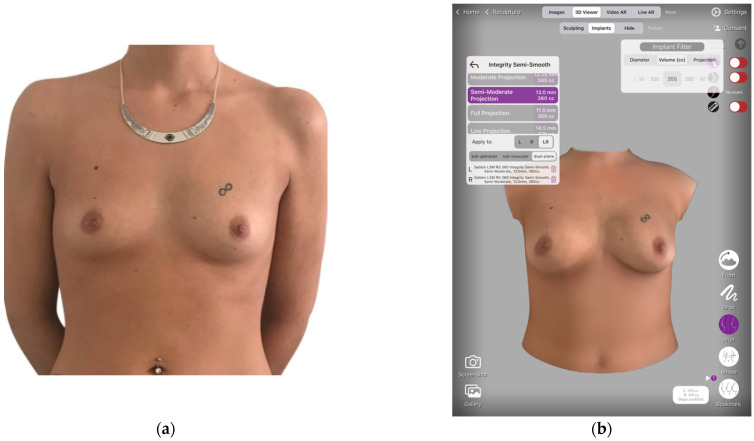

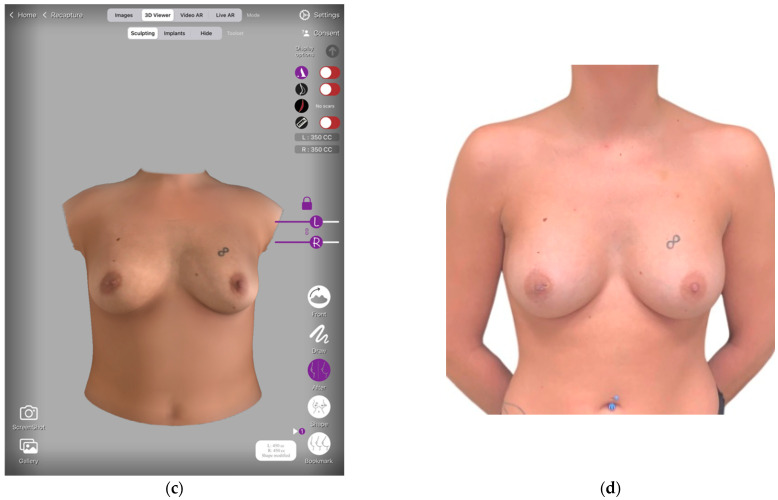
(**a**) Pre-operative photograph of a 27-year-old patient. (**b**) Breast augmentation simulation allows the patient to choose their breast implant, implant positioning (dual plane in this case), and surgical approach. (**c**) 3D breast augmentation simulation allows the provider to easily choose the implant volume with the patient. (**d**) Post-operative photograph of the same patient at the one-year follow-up.

**Figure 2:**
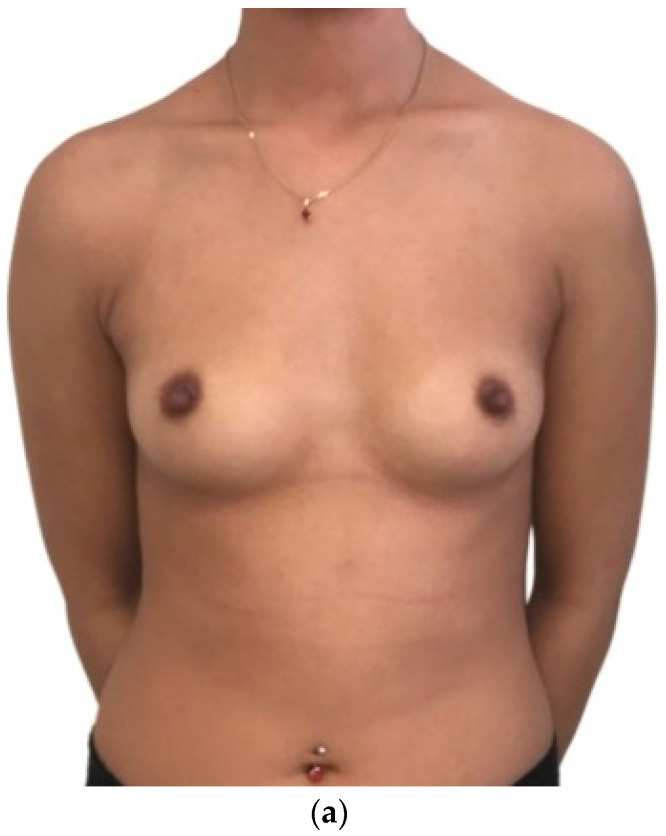

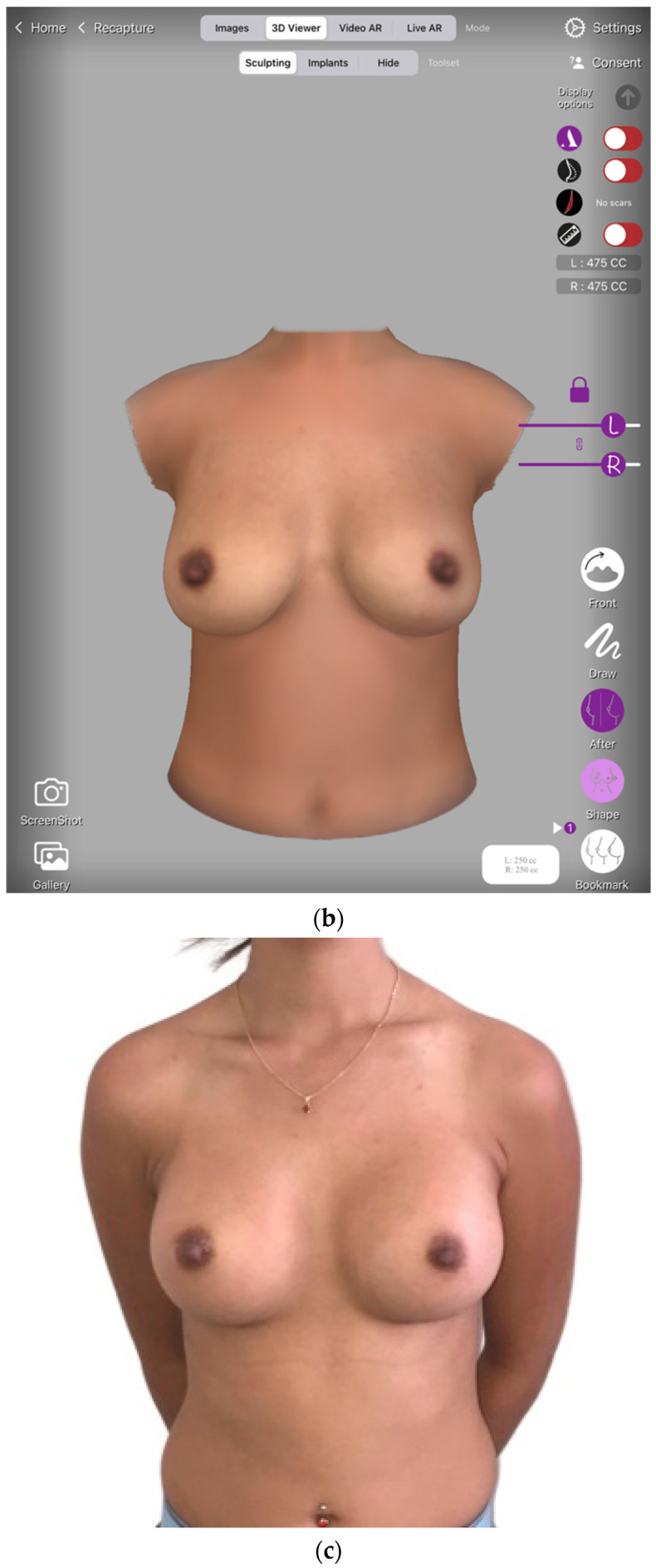
(**a**) Pre-operative photograph of a 25-year-old patient. (**b**) 3D breast augmentation simulation. Implant positioning was selected on a prepectoral plane. (**c**) Post-operative photograph of the same patient at the one-year follow-up.

All patients were satisfied with the simulations and the mean score on the Breast Simulation Assessment Scale (Table 1) was 3.4 ± 0.3 (Figure 1 and Figure 2). All patients were very satisfied with their breast volume with a mean VAS score of 8.2 ± 1.2.

The mean simulation time took 90 s on average, which significantly reduced the standard time taken to choose the breast implants.

## 4. Discussion

Worldwide, breast augmentation is the most common surgical procedure performed by plastic surgeons, representing 17.6% of all plastic surgical procedures [6]. Of the 1,862,506 breast augmentations performed worldwide in 2018, 17.3% were performed in the United States, followed by Brazil (14.8%), Mexico (3.8%), Germany (3.5%), Italy (3.5%), Argentina (2.7%), and Colombia (2.3%) [11]. A careful understanding of the patients’ wishes and desires remains of paramount importance in the surgical planning of breast augmentation. Recent developments have emerged that allow patients to better understand how they will look and feel post-operatively [12,13]. Computer imaging has also evolved, allowing patients to visualize how their breasts could look post-operatively [15,16]. The three-dimensional simulation of breast augmentation using Arbrea Breast Software appears to be useful for the pre-operative planning of breast augmentation. Our data demonstrate good and satisfying similarities between the simulations and post-operative outcomes. Our patients were very satisfied with the simulations and the final breast volume and shape. Collectively, these data suggest that ABS has great utility in the pre-operative planning of breast augmentation. To date, the only other software available for breast surgery simulation that has been studied is Crisalix.

Crisalix allows for live simulations, but it needs a 3D scanner to be plugged into the iPad. Arbrea Breast Software has some advantages over the Crisalix software (27): 

Arbrea Breast Software is:

1. Faster (clearly, as it takes a few seconds to take the pictures and less than one minute to generate the whole simulation).
2. Simpler to use (iPad only): the simplicity of Arbrea Breast Software does not only depend on it being an iPad-only solution. It was designed to be entirely and extremely simple to use and to not require any special training or learning curves. It is a plug-and-play solution with an incredible user interface (UI)/user experience (UX) that results in a process that takes less than 90 s and does not interfere with the doctor’s consultation process but instead simplifies it.
3. Safer (because the data stays on the device and does not go into the cloud).

From a technical point of view:

1. Because all computations are performed locally on the device, the whole process is more efficient and allows surgeons to operate in different consultation rooms, without even needing to connect to WiFi;
2. Because it works directly from RGB iPad images (or to put it simply, it works with photos rather than depth sensors, as in the case of Crisalix), it is the only tool, as far as we know, that can perform augmented reality simulations in online consultations directly on the patient’s body, as:
  - AR can be applied over an image;
  - AR can be applied over a video;
  - AR can be applied over a live consultation remotely with the patient.
3. It is almost entirely AI (artificial intelligence)-based, meaning that the algorithms were learned from the results of real patients.

Thanks to our study, we found ABS to be particularly useful in helping patients select between close implant volumes (i.e., 285 or 320 cc). This is important in the clinical practice since the final volume of the implant must be pre-operatively agreed upon between the patient and surgeon. Overall, our experience with ABS was positive and facilitated our pre-operative planning for breast augmentation. The present study was designed to assess the clinical utility and quality of simulations using ABS. Collectively, our results show that three-dimensional simulation using ABS has good clinical utility in pre-operative planning for breast augmentation. Further studies are needed to assess its efficacy for patients with breast ptosis as well.

## 5. Conclusions

Three-dimensional simulation is becoming increasingly common in pre-operative planning for breast augmentation. The present study aimed to assess the degree of similarity of the three-dimensional simulations generated using Arbrea Breast Software with the post-operative results. We found that ABS provides a very satisfying simulations for patients undergoing breast augmentation. However, we recommend informing patients that only the volume simulation is extremely accurate. On the other hand, it is necessary to not guarantee an absolute correspondence regarding the breast shape between the simulation and the post-operative result.

## Figures and Tables

**Table 1:** Breast augmentation simulation assessment scale developed by the first author (SLP).

How Close Is Your Simulation to the Real Result?
0 Totally different
1 Moderately different
2 Similar
3 Very similar
4 Identical

**Table 2:** Study data.

	Number
**Characteristic**	
**No. cases**	40
**Age, y**	
Mean (SD)	29.5 (3.1)
Minimum	18
Maximum	53
**BMI, kg/m^2^**	
Mean (SD)	25.8 (1.9)
Minimum	23
Maximum	29
**Comorbidities**	
Hypertension	2 (5%)
Hypothyroidism	1 (2.5%)
**Implant complications**	
Capsular contracture	1 (2.5%)
Palpable implant	1 (2.5%)

**Table 3:** Pre- and post-operative BREAST-Q values.

	Pre-Operative	Post-Operative	*p* Value
**Satisfaction with breasts**	13 ± 2.1	47 ± 3.1	0.005
**Psychological well-being**	29 ± 3.2	45 ±2.4	0.002
**Sexual well-being**	13.4 ± 4.1	21 ± 3.2	0.001
